# HIV-1 reverse transcriptase and protease mutations for drug-resistance detection among treatment-experienced and naïve HIV-infected individuals

**DOI:** 10.1371/journal.pone.0229275

**Published:** 2020-03-02

**Authors:** Farah Bokharaei-Salim, Maryam Esghaei, Khadijeh Khanaliha, Saeed Kalantari, Arezoo Marjani, Atousa Fakhim, Hossein Keyvani

**Affiliations:** 1 Department of Virology, School of Medicine, Iran University of Medical Sciences, Tehran, Iran; 2 Research Center of Pediatric Infectious Diseases, Institute of Immunology and Infectious Diseases, Iran University of Medical Sciences, Tehran, Iran; 3 Departments of Infectious Diseases and Tropical Medicine, Iran University of Medical Sciences, Tehran, Iran; 4 Department of Architectural Engineering, Faculty of Engineering, Islamic Azad University, South Tehran Branch, Tehran, Iran; University of Cincinnati College of Medicine, UNITED STATES

## Abstract

**Background:**

The presence of drug resistance mutations (DRMs) against antiretroviral agents is one of the main concerns in the clinical management of individuals with human immunodeficiency virus-1 (HIV-1) infection, especially in regions of the world where treatment options are limited. The current study aimed at assessing the prevalence of HIV-1 DRMs among naïve and treatment-experienced HIV-1-infected patients in Iran.

**Methods:**

From April 2013 to September 2018, the HIV-1 protease and reverse transcriptase genes were amplified and sequenced in plasma specimens of 60 newly diagnosed antiretroviral-naive individuals and 46 participants receiving antiretroviral therapies (ARTs) for at least six months with an HIV viral load of more than 1000 IU/mL to determine the HIV-1 DRMs and subtypes.

**Results:**

Among the 60 treatment-naïve HIV-1-infected participants, 8.3% were infected with HIV-1 variants with surveillance DRMs (SDRMs). The SDRMs, D67N and D67E, belonged to the NRTIs class in two patients and K103N and V106A belonged to the NNRTIs class in three patients. The phylogenetic analysis showed that 91.7% of the subjects were infected with subtype CRF35_AD, followed by subtype B (5.0%) and CRF01_AE (3.3%). Among the 46 ART-experienced participants, 33 (71.7%) carried HIV-1 variants with SDRMs (9.1% against PIs, 78.8% against NRTIs, and 100% against NNRTIs). M46I and I47V were the most common mutations for PIs, M184V was the most common mutation for the NRTIs, and K103N/S was the most common mutation for NNRTIs. Phylogenetic analysis of the polymerase region showed that all of the 46 HIV-1-infected patients who failed on ART carried CRF35_AD.

**Conclusions:**

The moderate prevalence of SDRMs (8.3%) in treatment-naïve and ART-failed (77.1%) Iranian patients with HIV-1-infection emphasizes the need for systematic viral load monitoring, expanding drug resistance testing, carefully surveilling individuals on ART regimens, and facilitating access to new antiretrovirals by health authorities.

## Introduction

Nearly 40 million individuals around the world are living with human immunodeficiency virus-1 (HIV-1) infection; more than half of them had access to antiretroviral therapies (ARTs) in 2017. However, thousands of people are newly infected with this virus each year [1].

The prevalence of HIV-1 infection remains low among the general population in Iran, but infection is highly prevalent among certain populations (e g, 13.8% in injecting drug users (IDUs). It is noteworthy that sexual transmission increased in recent years in Iran [2].

The introduction of ARTs since the 1990s significantly reduced the mortality and morbidity of the HIV-infected patients [3]. Currently, there are six various classes of antiretrovirals to treat HIV-1 infection. The most common ARTs in Iran are nucleoside/nucleotide reverse transcriptase inhibitors (NRTIs), non-nucleoside reverse transcriptase inhibitors (NNRTIs), and protease inhibitors (PIs) [4]. All newly diagnosed patients in Iran are currently receiving ARTs including two NRTIs (Zidovudine [AZT] and Lamivudine [3TC]) and one NNRTI (Efavirenz [EFV]) or one integrase inhibitor as the first-line therapy. When the treatment fails, PIs are added to ART as the second-line ART regimen [3, 5].

Regardless of the remarkable success in the treatment of HIV-1 infection, there is increasing concern about the emergence of HIV-1 drug resistance mutations (DRMs), which can lead to treatment failure [5]. HIV-1 drug resistance can be transmitted when patients carry HIV-1 variants with DRMs; the resistance can also be acquired when the patient is on ART regimen [5, 6].

In Europe, North America, and Brazil, the prevalence of HIV-1 drug resistance is 5%-15% in newly diagnosed individuals and 10%-25% in treatment-experienced patients [7, 8]. Therefore, it seems that the assessment of the prevalence of HIV-1 drug resistance can provide valuable information for clinicians before starting treatment, as well as in switching ART regimens when treatment failure is suspected [9]. The present study aimed at determining the prevalence of HIV-1 DRMs among treatment-experienced and treatment-naïve Iranian patients with HIV-1 infection.

## Patients and methods

### Study population

The current cross sectional study was conducted on 60 newly diagnosed antiretroviral (ARV) treatment-naive patients with HIV-1 infection (HIV Ag/Ab and HIV-RNA positive), and 592 HIV-infected patients receiving ART for more than six months referring to clinics and hospitals in Tehran, Iran from April 2013 to September 2018.

Among the patients receiving antiviral drugs for more than six months, 51 had a viral load of above 1000 IU/mL. According to the definition, people with HIV viral load of more than 1000 IU/mL for at least six months after ART initiation are probably infected with a drug-resistant HIV strain [10]. These patients were selected in the present study for HIV drug resistance testing. It should be noted that five patients did not continue their collaboration and left the study. Therefore, the study was performed using blood samples of 46 participants. The ART regimen of the 46 patients included NNRTI-based regimen for 37 patients (80.4%), PI-based regimen for eight patients (17.4%), integrase-based regimen for none of the subjects (0.0%), and mixed regimen for one patient (2.2%).

### Ethical considerations

The current study protocol was approved by the Research Ethics Committee of School of Medicine at Iran University of Medical Sciences, Tehran, Iran (ethical code: IR. IUMS. FMD.REC 1396.28765); all the experiments and procedures were in accordance with the principles of the Declaration of Helsinki and the Iranian National Ethical Guidelines for Biomedical Research.

The participants were informed about the study objectives and written consent was obtained from all of them before enrolment.

### Collection of the specimens and processing

A 4-mL peripheral blood sample obtained from each participant was drained into a sterile vacutainer tube containing EDTA (ethylenediaminetetraacetic acid). The plasma of all specimens was separated from the whole blood and kept at ‒80°C utile use. The HIV-1 viral load of participants was measured using a method described elsewhere [11]. The plasma samples of eight patients with HIV infection and eight healthy participants were used as positive and negative controls, respectively.

### Detection of HIV-1 DRMs

To detect HIV DRMs, the viral RNA was isolated from 140 μL of the plasma specimen using the QIAamp Viral RNA Extraction kit (Qiagen GmbH, Hilden, Germany) according to the manufacturer’s instructions. The quantity and quality of the extracted RNAs were determined using a NanoDrop^™^ spectrophotometer (Thermo Scientific, Wilmington, USA). Then, the encoding region of the HIV-1 protease gene (i e, PR, HXB2 position 2358–2550) and the N-terminal region of the reverse transcriptase gene (i e, RT, HXB2 position 2673–3269) were amplified by specific primers (forward primer [K1] and reverse primer [U13]) [12]. The primers used in the present study are shown in Table 1. The SuperScript III One-Step RT-PCR System with Platinum *Taq* High Fidelity (Invitrogen, Carlsbad, CA, USA) was used in the first stage of polymerase chain reaction (PCR) and complementary DNA (cDNA) synthesis. Then, the PCR product was re-amplified with inner primers (forward primer [K4] and reverse primer [U12]) with LA *Taq* DNA polymerase (TaKaRa Bio Inc., Shiga, Japan),[13, 14]. The HIV-1 DRMs were detected according to the method described previously [11].

**Table 1:** Primers used to detect HIV-1 drug resistance mutants.

**Primers:**			
**Application**		**Name**	**Primer Sequences for Pol Gene**
**cDNA Synthesis and PCR first run**	**Forward-1**	**K1**	**5'-AAG GGC TGT TGG AAA TGT GG-3'**
	**Reverse-1**	**U13**	**5'-CCC ACT CAG GAA TCC AGG T-3'**
**Nested-PCR**	**Forward-2**	**K4**	**5'-GAA AGG AAG GAC ACC AAA TGA-3'**
	**Reverse-2**	**U12**	**5'-CTC ATT CTT GCA TAT TTT CCT GTT-3'**
**Primers for Sequencing**			
**Nested-PCR Protease region**	**Forward**	**DRPRO1M**	**5'-AGA GCC AAC AGC CCC ACC AG-3'**
	**Reverse**	**DRPRO6**	**5'-ACT TTT GGG CCA TCC ATT CC-3'**
**Nested-PCR (reverse transcriptase region**	**Forward**	**DRRT7L**	**5'-GAC CTA CAC CTG TCA ACA TAA TTG G-3'**
	**Reverse**	**DRRT6L**	**5'-TAA TCC CTG CAT AAA TCT GAC TTG C-3'**

### Analysis of sequences

**Fig 1:**
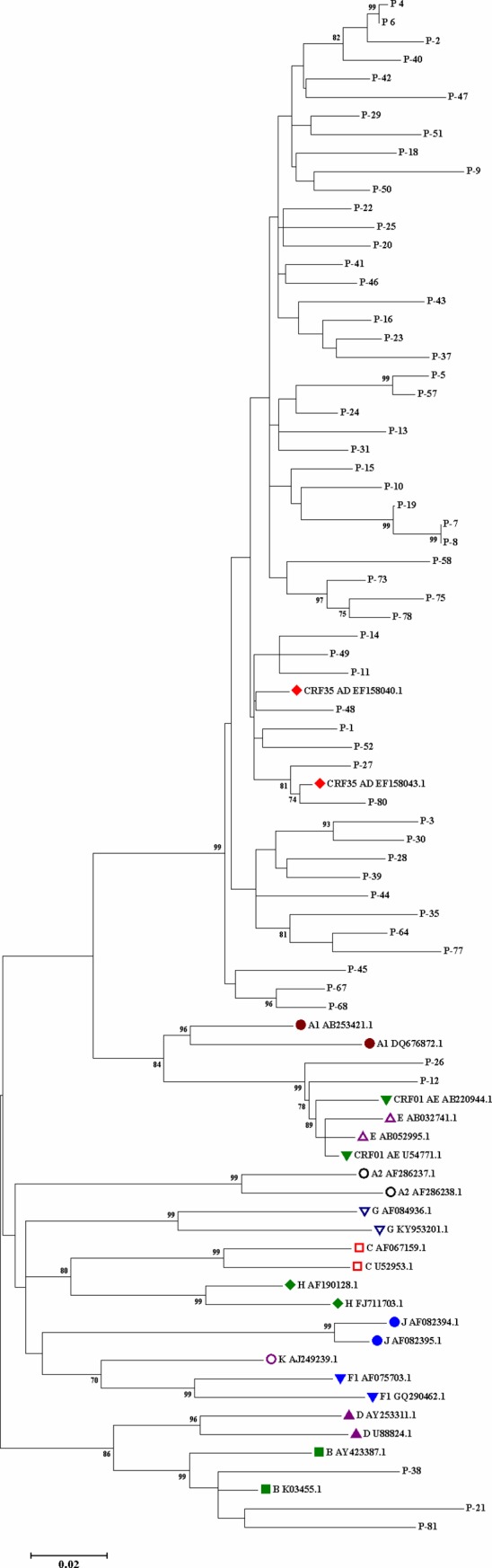
Phylogenetic tree was dawned using MEGA7 software based on HIV-1 protease and reverse transcriptase nucleotide sequences (1015 bp) obtained from plasma samples of 60 treatment-naïve Iranian patients with HIV-1 infection and those corresponding to various HIV subtypes/CRFs obtained from the GenBank HIV database. The Phylogenetic tree was constructed using the neighbor-joining method; the bootstrap values over 70% obtained after 1000 replicates are also shown.

**Fig 2:**
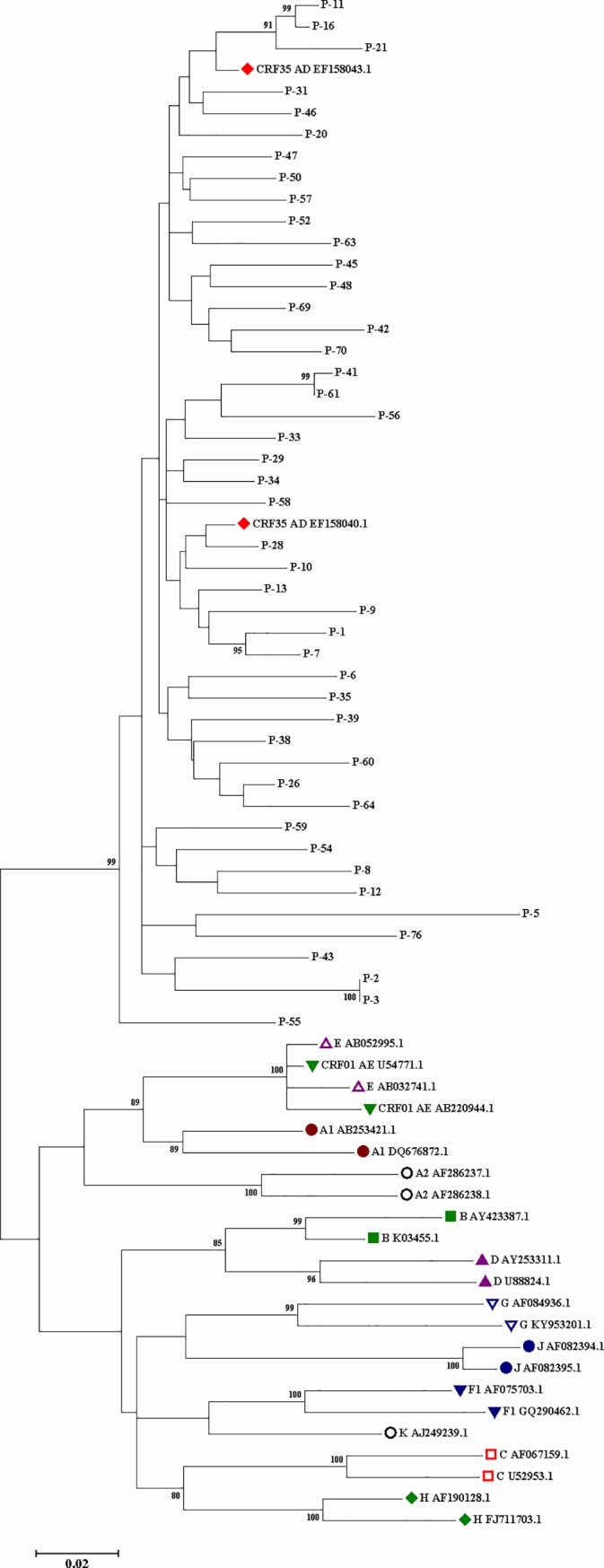
Phylogenetic tree was dawned using MEGA7 software based on HIV-1 protease and reverse transcriptase nucleotide sequences (1015 bp) obtained from plasma samples of 46 treatment-experienced Iranian patients with HIV infection and those corresponding to various HIV subtypes/CRFs obtained from the GenBank HIV database. The phylogenetic tree was constructed using the neighbor-joining method; the bootstrap values over 70% obtained after 1000 replicates are also shown.

The obtained sequences were aligned with HIV-1 reference sequence (GenBank accession number: K03455) by the CLC Main Workbench 5.5 software (CLCbio, Boston, MA, USA). The drug resistance mutations were determined using the list of surveillance drug-resistant mutations (SDRMs) and also DRMs developed by the World Health Organization (WHO) [15]. Two phylogenetic trees were constructed by the neighbor-joining method using MEGA software (version 7.0.21) (Fig 1 and Fig 2). The statistical significance of the phylogenetic tree was evaluated using the bootstrap method (1000 replicates).

### Nucleotide sequence accession numbers

The nucleotide sequences of the HIV-1 protease and reverse transcriptase (PR-RT) regions obtained from treatment-naïve and treatment-experienced HIV-infected Iranian individuals (1015 bp) were submitted to GenBank and are already available under the accession numbers KY816748 to KY816750, KX641030 to KX641071, MK318950 to MK318964, and MK302501 to MK302546.

### Statistical analysis

The data analysis was performed using SPSS version 20 software (SPSS Inc., Chicago, IL). The normality of the quantitative variables was evaluated by the Kolmogorov–Smirnov test. The analysis of continuous variables was conducted using the Kruskal–Wallis test. The Fisher exact and Chi-squared tests were applied to assess the statistical differences between the two groups. A P-value of ≤0.05 was considered statistically significant.

## Results

### Characteristics of participants

A sample of 60 HIV-1-infected ART-naïve (males: 63.0%) with a mean age of 32.8 ± 11.0 years (ranged: 2–60) was enrolled in the current cross sectional study. Significant associations were observed between the gender, and CD4 count (*P* = 0.019) and epidemiological parameters including history of intravenous drug use (*P* = 0.004), sex partner of injecting drug user (*P*< 0.001), and history of imprisonment (*P* = 0.004) in the treatment-naïve HIV-infected patients.

Moreover, 592 patients with established HIV-1 infection (males: 65.9%) receiving ART for more than six months were enrolled, among whom 51 (8.6%) had a viral load of above 1000 IU/mL. Of these 51 patients, five did not continue their collaboration and left the study; therefore, the plasma specimens of 46 participants were assessed. The demographic characteristics and laboratory findings of the patients are summarized in Table 2. They had a mean age of 39.9 ± 10.5 years (ranged: 1–70) years. Their mean CD4 count was 517.5±311.6 (ranged 16–3112); 405 (68.3%) having CD4 counts of less than 350. A significant association was observed between the HIV viral load of ±1000 IU/mL, and gender (*P* = 0.046), CD4 count (*P* <0.001) and epidemiological characteristics such as the history of imprisonment (*P* = 0.002), history of tattooing (*P* = 0.001), intravenous drug use (*P* = 0.002), sex partner of injecting drug user (*P* = 0.047), and mother to child transmission (Table 2). A significant association was also observed between the HIV viral load of ±1000 IU/mL and the level of education (*P* = 0.007) (Table 3). The demographic and epidemiological characteristics of the studied participants are shown in Table 3.

**Table 2:** Demographic characteristics and laboratory findings of patients with human immunodeficiency virus infection.

Parameter		Viral Load (IU/mL) <999	Viral Load (IU/mL) ≥1000	Total	*P*-value
**No. of Patients**	**Male**	**350 (67.4%)**	**40 (78.4%)**	**390 (65.9%)**	**0.046** Fisher Exact Test
	**Female**	**191 (35.3%)**	**11 (21.6%)**	**202 (34.1%)**	
**Age (yr) ± SD**		**40.2 ± 10.1 (1–70)**	**37.7 ± 13.8 (3–69)**	**39.9 ± 10.5 (1–70)**	**0.372** Mann-Whitney U
**Laboratory Parameters**					
**CD4 count**		**532.5 ± 309.1 (18–3112)**	**306.5 ± 295.8 (16–1446)**	**517.5 ± 311.6 (16–3112)**	**<0.001**^**a**^ Mann-Whitney U
**CD4 categorized**	**<349**	**162 (29.9%)**	**26 (50.0%)**	**188 (31.7%)**	**0.005**^**a**^ Fisher Exact Test
	**≥ 350**	**379 (70.1%)**	**26 (50.0%)**	**405 (68.3%)**	
**Viral Load IU/mL (Median)**		**57.5 ± 157.1 (0–996)**	**1192815.9 ± 2752035.3 (1,776–17,977,524)**	**104,650.1 ± 875,482.9 (0–17,977,524)**	**<0.001**^**a**^ Mann-Whitney U
**AST**^**1**^ **(IU/L)**		**31.6 ± 23.0 (5–211)**	**38.2 ± 35.1 (12–236)**	**32.2 ±24.4 (5–236)**	**0.025**^**a**^ Mann-Whitney U
**ALT**^**2**^ **(IU/L)**		**35.0 ± 26.2 (4–231)**	**30.3 ± 15.6 (7–88)**	**34.6 ± 25.5 (4–231)**	**0.538** Mann-Whitney U
**ALP**^**3**^ **(IU/L)**		**260.3 ± 113.9 (102–987)**	**290.4 ± 158.8 (102–890)**	**262.9 ± 118.7 (102–987)**	**0.317** Mann-Whitney U

1. Aspartate aminotransferase (AST)

2. Alanine aminotransferase (ALT)

3. Alkaline phosphatase (ALP)

^a^. Statistically significant

**Table 3:** Demographic and epidemiological characteristics of patients with human immunodeficiency virus infection.

Parameters		Viral Load (IU/mL) <999	Viral Load (IU/mL) ≥1000	Total	*P*-value
**No. of Patients**	**Male**	**350 (67.4%)**	**40 (78.4%)**	**390 (65.9%)**	**0.046** Fisher Exact Test
	**Female**	**191 (35.3%)**	**11 (21.6%)**	**202 (34.1%)**	
**Age (yr) ± SD**		**40.2 ± 10.1 (1–70)**	**37.7 ± 13.8 (3–69)**	**39.9 ± 10.5 (1–70)**	**0.372** Mann-Whitney U
**Epidemiological Characteristics**					
**History of imprisonment**		**162 (29.9%)**	**27 (51.9%)**	**189 (31.9%)**	**0.002**^**a**^ Fisher Exact Test
**History of partner imprisonment**		**38 (7.0%)**	**3 (5.8%)**	**41 (6.9%)**	**1.000** Fisher Exact Test
**History of tattooing**		**106 (19.6%)**	**21 (40.4%)**	**127 (21.4%)**	**0.001**^**a**^ Fisher Exact Test
**History of blood transfusion**		**25 (4.6%)**	**2 (3.8%)**	**27 (4.6%)**	**1.000** Fisher Exact Test
**Intravenous drug user**		**259 (47.9%)**	**37 (71.2%)**	**296 (49.9%)**	**0.002**^**a**^ Fisher Exact Test
**Sexual partner of injecting drug user**		**114 (21.1%)**	**5 (9.6%)**	**119 (20.1%)**	**0.047**^**a**^ Fisher Exact Test
**History of surgery**		**82 (15.2%)**	**6 (11.5%)**	**88 (14.8%)**	**0.682** Fisher Exact Test
**History of unprotected sexual contact**		**288 (53.2%)**	**25 (48.1%)**	**313 (52.8%)**	**0.561** Fisher Exact Test
**History of needle stick**		**57 (10.5%)**	**5 (9.6%)**	**62 (10.5%)**	**1.000** Fisher Exact Test
**Mother to child transmission**		**9 (1.7%)**	**5 (9.6%)**	**14 (2.4%)**	**0.005** Fisher Exact Test
**Education**	**Incomplete high school diploma**	**343 (63.4%)**	**43 (82.7%)**	**386 (65.1%)**	**0.007**^**2**^ Chi-Square Test
	**High school diploma**	**134 (24.8%)**	**7 (13.5%)**	**141 (23.8%)**	
	**Above high school diploma**	**63 (11.6%)**	**2 (3.8%)**	**65 (11.0%)**	
**Marital Status**	**Single**	**149 (27.5%)**	**21 (40.4%)**	**170 (28.7%)**	**0.411** Chi-square test
	**Married**	**287 (53.0%)**	**18 (34.6%)**	**305 (51.4%)**	
	**Divorced**	**66 (12.2%)**	**11 (21.2%)**	**77 (13.0%)**	
	**Widowed**	**37 (6.8%)**	**2 (3.8%)**	**39 (6.6%)**	

^a^. Statistically significant

After evaluation of the phylogenetic trees, it was found that some sequences clustered closely and looked identical: patients 7 and 8 in the phylogenetic tree #1 and patients 2 and 3 in phylogenetic tree #2. A review of the patients' files revealed that these people were infected from a same source, and they were family members. To verify the finding of similar sequences in certain individuals, a second PCR was run to rule out cross-contamination of samples.

### Surveillance drug-resistant mutations and HIV-1 subtypes in treatment-naïve HIV-1-infected individuals

Among the 60 treatment-naïve HIV-1-infected participants, the PR-RT amplified sequences were aligned with HIV-1 reference sequences belonging to subtypes/CRFs obtained from the Los Alamos Sequence Database (http://www.hiv.lanl.gov/) using MEGA software (version 7.0.21). The phylogenetic tree was constructed by Molecular Evolutionary Genetics Analysis version 7 (MEGA7) software and the result is shown in Fig 1. The phylogenetic analyses of the PR-RT region of HIV-1 showed that CRF35_AD accounted for 91.7% (55/60) of the HIV-1-infected patients, followed by subtype B with 5.0% (3/60) and CRF01_AE with 3.3% (2/60).

Overall, 8.3% of the subjects were infected with HIV-1 variants with SDRMs according to the last WHO algorithm. In the present study, SDRMs belonged to the NRTIs class including D67N and D67E, which were found in two patients. D67N is a non-polymorphic thymidine analog mutation (TAM) associated with low-level resistance to AZT and Stavudine (D4T). When accompanied by other TAMs, it leads to reduced susceptibility to Abacavir (ABC), Didanosine (DDI), and Tenofovir (TDF). D67E is a non-polymorphic mutation that generally occurs in viruses with multiple TAMs [16, 17].

It was also observed that the SDRMs belonged to the NNRTIs class including K103N and V106A detected in three patients. K103N is a non-polymorphic mutation that induces high-level reductions in the susceptibility to Nevirapine (NVP) and Efavirenz (EFV). V106A is a non-polymorphic mutation that can cause high-level resistance to Nevirapine (NVP) and intermediate resistance to Efavirenz (EFV). This mutation is selected in vivo and in vitro by Doravirine (DOR) and it alone can lead to an intermediate decline in DOR susceptibility. V106A is associated with high-level resistance to DOR when accompanied by other DOR-associated DRMs. Three minor HIV protease inhibitor-related mutations (L10I, L10V, and G73S) were also detected in four patients, although these mutations are not included in the WHO SDRMs list (Table 4).

**Table 4:** Frequency of human immunodeficiency virus surveillance drug resistance mutations in treatment-naïve Iranian patients with HIV infection.

SDRMs Based on WHO List	No (%)	HIV-1 Subtype
**NRTI^1^-resistance Mutations**		
**K65I**	**1 (1.7)**	**CRF35_AD**
**D67E**	**1 (1.7)**	**CRF35_AD**
**D67N**	**1 (1.7)**	**CRF35_AD**
**K70I**	**1 (1.7)**	**CRF35_AD**
**T215N**	**2 (3.3)**	**CRF35_AD**
**NNRTI**^2^**-resistance Mutations**		
**E138A**	**1 (1.7)**	**CRF35_AD**
**K103N**	**3 (5.0)**	**CRF35_AD**
**V106A**	**1 (1.7)**	**CRF35_AD**
**V106I**	**4 (6.7)**	**B (1 patients) CRF35_AD (3 patients)**
**Y181N**	**1 (1.7)**	**CRF35_AD**
**Major PI**^3^**-resistance Mutations None**		
**Minor PI-resistance Mutations (based on IAS-USA list)**		
**G73S**	**1 (1.7)**	**CRF35_AD**
**L10I**	**2 (3.3)**	**B (1 patient) CRF35_AD (1 patient)**
**L10V**	**1 (1.7)**	**CRF35_AD**

1. Nucleoside reverse transcriptase inhibitors

2. Non-nucleoside reverse transcriptase inhibitors

3. Protease inhibitors

### Drug-resistant mutations and HIV-1 subtypes in HIV-1-infected patients who failed on ART

The PR-RT obtained sequences of the plasma samples of 46 HIV-1 positive participants that were on ART for at least six months and had an HIV viral load of above 1000 IU/mL after six months of receiving ARTs were aligned with HIV-1 reference sequences corresponding to nine subtypes/CRFs of HIV-1 retrieved from the Los Alamos Sequence Database (http://www.hiv.lanl.gov/) using Clustal W, followed by Molecular Evolutionary Genetics Analysis (MEGA 7) (Fig 2). Phylogenetic analyses of the polymerase region showed that all 46 (100%) studied participants were infected with CRF35_AD.

From all patients who failed on ARTs, 28.2% (13/46) did not carry any drug resistance mutation. However, 33 (71.7%) patients carried HIV-1 variants with DRMs, including three (9.1%) patients against PIs, 26 (78.8%) against NRTIs, and 33 (100%) against NNRTIs. The level of resistance to 20 antiretroviral drugs among HIV-1-infected patients who failed on treatment is summarized in Table 5.

**Table 5:** The level of resistance to antiretroviral drugs among treatment-failed patients with HIV-1 infection.

Name	Susceptible	Low-Level Resistance	Potential Low-Level Resistance	Intermediate Resistance	High-Level Resistance
**Protease Inhibitors: 3 (6.4%) Patients**					
**Atazanavir/r (ATV/r)**	44 (93.6%)	1 (2.1%)	_	1 (2.1%)	1 (2.1%)
**Darunavir/r (DRV/r)**	45 (95.7%)	_	_	1 (2.1%)	1 (2.1%)
**Fosamprenavir/r (FPV/r)**	44 (93.6%)	_	1 (2.1%)	_	2 (4.3%)
**Indinavir/r (IDV/r)**	44 (93.6%)	1 (2.1%)	_	1 (2.1%)	1 (2.1%)
**Lopinavir/r (LPV/r)**	44 (93.6%)	1 (2.1%)	_	1 (2.1%)	1 (2.1%)
**Nelfinavir (NFV)**	44 (93.6%)	1 (2.1%)	_	_	2 (4.3%)
**Saquinavir/r (SQV/r)**	44 (93.6%)	1 (2.1%)	1 (2.1%)	1 (2.1%)	_
**Tipranavir/r (TPV/r)**	44 (93.6%)	1 (2.1%)	_	2 (4.3%)	_
**Nucleoside Reverse Transcriptase Inhibitors: 26 (55.3%) Patients**					
**Abacavir (ABC)**	21 (44.7%)	13 (27.7%)	5 (10.6%)	8 (17.0%)	_
**Zidovudine (AZT)**	34 (72.3%)	3 (6.4%)	4 (8.5%)	6 (12.8%)	_
**Stavudine (D4T)**	32 (68.1%)	2 (4.3%)	5 (10.6%)	8 (17.0%)	_
**Didanosine (DDI)**	21 (44.7%)	14 (29.8%)	5 (10.6%)	_	_
**Emtricitabine (FTC)**	22 (46.8%)	_	24 (51.1%)	_	1 (2.1%)
**Lamivudine (3TC)**	22 (46.8%)	_	24 (51.1%)	_	1 (2.1%)
**Tenofovir (TDF)**	36 (76.6%)	6 (12.8%)	5 (10.6%)	_	_
**Non-nucleoside Reverse Transcriptase Inhibitors: 33 (70.2%) Patients**					
**Doravirine (DOR)**	22 (46.8%)	7 (14.9%)	11 (23.4%)	7 (14.9%)	_
**Efavirenz (EFV)**	16 (34.0%)	_	2 (4.3%)	29 (61.7%)	_
**Etravirine (ETR)**	27 (57.4%)	11 (23.4%)	7 (14.9%)	2 (4.3%)	_
**Nevirapine (NVP)**	16 (34.0%)	_	_	31 (66.0%)	_
**Rilpivirine (RPV)**	27 (57.4%)	7 (14.9%)	2 (4.3%)	11 (23.4%)	_

The most common mutations were M46I and I47V for PIs, M184V for NRTIs, and K103N/S for NNRTIs (Table 6). The frequencies of different DRMs among ART-failed Iranian patients, based on the Stanford HIV Drug Resistance Database are shown in Table 6. These patients had various levels of sensitivity to 20 antiretroviral drugs (Table 5).

**Table 6:** Frequency of drug resistance mutations among treatment-failed Iranian patients with HIV, based on WHO List.

Class of Medication	PIs^1^-resistance Mutations		NRTIs^2^resistance Mutations		NNRTIs^3^-resistant Mutations	
	Name	Number	Name	Number	Name	Number
**Type of mutation**	**Major Resistance Mutations**		**M**41**L**	**4**	**A**98**G**	**2**
	**V**32**I**	**1**	**K**65**R/E**	**3**	**L**100**V**	**1**
	**M**46**I**	**2**	**D**67**N/G**	**6**	**K**101**E/R**	**5**
	**I**47**V**	**2**	**K**70**R**	**6**	**K**103**N/S**	**19**
	**I**50**L**	**1**	**L**74**V**	**1**	**V**106**M**	**1**
	**I**54**L/V**	**2**	**V**75**M**	**8**	**V**108**I**	**4**
	**L**76**V**	**1**	**F**77**L**	**2**	**E**138**G/A**	**2**
	**PI Accessory Resistance Mutations**		**Y**115**F**	**1**	**V**179**T**	**1**
	**L**10**F**	**2**	**M**184**V**	**25**	**Y**181**C**	**2**
	**K**20**T**	**1**	**L**210**W**	**1**	**Y**188**L/H**	**5**
	**L**33**F**	**1**	**T**215**F/I/Y**	**9**	**G**190**A/S**	**10**
	**Q**58**E**	**1**	**K**219**E/Q/R**	**6**	**H**221**Y**	**1**
	**L**89**V**	**1**	_	_	**P**225**H**	**10**
	_	_	_	_	**M**230**L**	**1**
	_	_	_	_	**P**236**L**	**1**
	_	_	_	_	**K**238**T/N**	**2**
	_	_	_	_	**N**348**I**	**1**

1. Protease inhibitors

2. Nucleoside reverse transcriptase inhibitors

3. Non-nucleoside reverse transcriptase inhibitors

## Discussion

The detection of primary and secondary ART resistance provides valuable information that is critical to determine ART regimens [5]. In the present study, the overall prevalence of SDRMs was 8.3% among 60 treatment-naïve HIV-1-infected patients. Phylogenetic analysis showed that CRF35_AD accounted for 91.7% of these patients, followed by subtype B with 5.0% and CRF01_AE with 3.3%. From the 46 treatment-failed participants, 33 (71.7%) patients carried HIV-1 variants with DRMs [three (9.1%) patients against PIs, 26 (78.8%) patients against NRTIs, and 33 (100%) patients against NNRTIs]. Phylogenetic analysis of the pol region showed that all 46 patients were infected with CRF35_AD.

The SDRM prevalence in various geographical areas is classified by the WHO as low (<5%), moderate (5%-15%), and high (>15%) [18]. This categorization shows the level of HIV Drug Resistance Surveillance needed to monitor primary HIV-DR [3]. In the current study, the prevalence of SDRMs was 8.3% in ART-naïve subjects; therefore, the study subjects are classified in the WHO moderate category in terms of the presence of SDRMs. The present study findings were consistent with those of the previous studies in Iran (primary resistance of 5%-15%) [11, 19, 20]. This finding was not surprising considering that NRTIs are available since 1997 and widely distributed in Iran as an essential part of ART regimens. Mutations conferring resistance to NRTIs and NNRTIs are the most prevalent forms of drug resistance detected globally, while PI resistance is universally less frequent [21].

According to a meta-analysis by Gupta et al., the prevalence of HIV-1 drug resistance to NNRTIs in treatment-naïve individuals in 2016 was 10.1% in Eastern Africa, 11.0% in Southern Africa, 9.4% in the Caribbean and Latin America, and 7.2% in Western and Central Africa. In the mentioned study, the increases in the rate of treatment-naïve patients with HIV-infection varied from 0.3% in Asia to 1.8% in Southern Africa from 2015 to 2016 [22].

In the present study, the prevalence of primary resistance among ART-naive Iranian patients (8.3%) was lower than that of many other countries [Germany (17.2%) [23], Greece (16.9%) [24], Brazil (13.3%) [25], and France (10.8%) [26]], but it seems to be rising when compared to previous reports from Iran [11, 19, 20, 27].

It is reported that when the frequency of transmitted drug resistance in a region is 8%-10%, drug resistance testing is cost-effective before the initiation of ART regimens in HIV-1-infected patients [8]. In the current study, HIV-1 SDRMs (D67N and D67E) belonging to the NRTIs class were found in two subjects and SDRMs) K103N and V106A (belonging to the NNRTIs class were found in three subjects.

From the 46 treatment-failed patients, 28.2% (13 patients) did not show any drug resistance mutation. However, 33 (71.7%) patients carried HIV-1 variants with DRMs [three (9.1%) patients against PIs, 26 (78.8%) patients against NRTIs, and 33 (100%) patients against NNRTIs]. M46I and I47V were the most frequent mutations for PIs; M184V was the most common mutation for the NRTIs, and K103N/S was the most common mutation for NNRTIs.

The prevalence of HIV-1 drug resistance in patients who failed on ART was 36.4% in Pakistan [28], 37.0%–45.0% in Switzerland [29], 56.3% in France [30], 52.0%-61.0% in China [31, 32], 75.5% in Taiwan [33], 71.1% in Ghana [14], 98.0% in Sub-Saharan Africa (13 clinics in Kenya, Malawi, Uganda, and Zimbabwe) [34]. Moreover, 42.0%-86.2% of the HIV-infected individuals had at least one resistance mutation in Iran [5, 9, 20]. It was also reported in China that with the increase in the coverage of ART, drug resistance increased from 23.0% to 74.0% from 2010 to 2016 [35].

According to the results of the present study and previous reports from Iran, it appears that more than half of the patients receiving antiretroviral therapy have evidence of DRMs [5, 9, 20]. Therefore, this issue deserves to be addressed in the future.

It is noteworthy that one of the critical factors to prevent emerging HIV drug resistant variants, and also inhibit virologic failure is patients’ adherence to ART [4, 36]. In the current study, female and male patients represented various values in terms of HIV viral load and DRMs. The HIV viral load of more than 1000 IU/mL was observed in 21.6% of females and 78.4% of males; in addition, HIV-1 DRM level in patients who failed on ART was lower in females (12.1% of females and 87.9% of males). This can be due to the higher compliance of female patients to ART than males.

Phylogenetic analysis of the pol region of HIV-1 showed that all the 46 (100%) treatment-failed participants were infected with CRF35_AD. There are several reports on the presence of various HIV-1 subtypes and CRFs (CRF35_AD, B, C, A, CRF35_AE, 45_CPX) in Iran [5, 9, 11, 19–21, 37, 38]. In the present study, it was found that ART-naive Iranian patients were infected with CRF35_AD (91.7%), subtype B (5.0%), and CRF01_AE (3.3%). Interestingly, in the current study, all of the treatment-failed patients were infected with CRF35_AD. Therefore, the result of the study disagrees with other reports from Iran.

It should be noted that ARTs are developed mainly using HIV-1 reference virus (subtype B), and in vitro studies suggested that various HIV-1 subtypes may affect the sensitivity of certain ARTs [39, 40]. It is reported that various HIV-1 subtypes respond differently to ARTs [41, 42]. Several studies from Africa show that the progression of the disease is higher in people infected with HIV-1 subtype D [43–45]. It is noteworthy that so far, no research is conducted in Iran on the response to various HIV-1 subtypes. Therefore, different subtypes may respond differently to ARTs. It seems that this issue needs further investigations on HIV-1 infected patients with larger sample sizes.

The appropriate access to ART regimens since 2003 reduced HIV-1-related morbidity and mortality in Iran. However, this success may be threatened by the widespread occurrence of both transmitted and acquired HIV-1 drug resistance to antiretroviral drugs [20]. Currently, most naïve HIV-1-infected Iranian patients undergo ARTs without performing drug resistance testing. Therefore, according to the results of the present and previous studies conducted in Iran, it seems that performing HIV-1 drug resistance tests before the onset of ARTs and the change of ART regimens (when treatment failure is suspected) can provide helpful information for physicians for the successful treatment of HIV-1-infected patients [9].

## Conclusions

The current study illustrated that the prevalence of SDRMs in the treatment-naïve Iranian patients with HIV-infection was 8.3%. SDRMs (D67N and D67E) belonging to the NRTIs class were found in two subjects and SDRMs) K103N and V106A (belonging to the NNRTIs class were found in three subjects. On the other hand, the frequency of DRMs was 71.7% in treatment-failed patients with HIV-1 infection; DRMs belonging to PIs were found in 9.1%, NRTIs in 78.8%, and NNRTIs in 100% of the patients. Therefore, due to the increasing drug resistance in treatment-naïve and treatment-failed patients with HIV in Iran, it seems essential to perform drug resistance testing to detect SDRMs and DRMs in such patients, respectively.

## Supporting information

S1 File (RAR)
